# Statistical Texture Modeling for Medical Volume Using Linear Tensor Coding

**DOI:** 10.1155/2013/630902

**Published:** 2013-06-25

**Authors:** Junping Deng, Xu Qiao, Yen-Wei Chen

**Affiliations:** ^1^College of Information Science and Engineering, Ritsumeikan University, Kusatsu 5250072, Japan; ^2^School of Control Science and Engineering, Shandong University, Jinan 250100, China; ^3^College of Computer Science and Information Technology, Central South University of Forestry and Technology, Hunan 410004, China

## Abstract

We introduced a compact representation method named Linear Tensor Coding (LTC) for medical volume. With LTC, medical volumes can be represented by a linear combination of bases which are mutually independent. Furthermore, it is possible to choose the distinctive basis for classification. Before classification, correlations between category labels and the coefficients of LTC basis are used to choose the basis. Then we use the selected basis for classification. The classification accuracy can be significantly improved by the use of selected distinctive basis.

## 1. Introduction

In the recent years, the research of digital atlases is a popular and important topic in the medical volume processing [1, 2]. Many problems in medical volumes interpretation involve the need of a modeling to understand the volumes with which it is presented, and thus well representation of medical volumes is very important part of computer-assisted diagnosis (CAD). Due to much variability in biological structures, it makes medical volume interpretation to be a difficult task.

Currently the representation of medical volume can be mainly categorized as shape-based methods, in which a deformable model is represented or matched to, and appearance based methods, in which the model represents the volume region covered by the structures. Statistical shape model (SSM) can construct the generic structure (mean structure) and deformation for a shape ensemble [3]. Due to the deformation of the organ shape in some special disease, it is widely utilized in medical image processing, such as medical image registration and segmentation [4, 5]. Inspired by the work of active shape model (ASM), 3D ASM was proposed for construction of 3D statistical models for segmentation of the left ventricle of the heart [6]. The statistical shape models also show good performance for distinguishing the abnormal liver from the normal one in [7]. Because many diseases change the texture (voxel value) of the organ significantly, we need to capture not only shape variations, but also texture (voxel value) variations. So the active appearance model (AAM) is proposed which can represent both shape and texture information. In [8], 3D active appearance model is used for segmentation of cardiac MR and ultrasound images. It is also possible to combine the two approaches together. For example, Mitchell et al. [9] used a combination of ASM and AAM to segment cardiac images. At each iteration, the two models ran independently to compute new estimates of the pose and shape parameters. For diagnosis assistance, making an accuracy diagnosis decision of liver is important for patient. Radiologists are mainly depending on the intensity variations (texture information) in livers on medical images to identify modules or tumors and make a diagnostic decision. However, there has been little research on applications of digital atlas to CAD. 

Compared to statistical shape modeling, statistical texture modeling usually faces overfitting problems, and the statistical texture modeling for medical volumes is a challenging task because the dimensions of the medical volume are very high, while the training samples are fewer than the dimensions of the data. In [10], we have proposed generalized N-dimensional principal component analysis (GND-PCA) for the modeling of a series of medical volumes. It is able to achieve good performance on construction of statistical appearance models for medical volumes with few samples. The medical volume is treated as a 3rd-order tensor, and the optimal subspace on each mode is calculated simultaneously by minimizing the square error between the original volumes based on the subspace with an iteration algorithm. However, this method has some disadvantages, such as each basis of the GNDPCA not being independent and thus making the core tensor of the final result redundant. Also it is difficult to choose the distinctive basis for classification.

To resolve the these problems, in our previous work, we proposed a linear tensor coding (LTC) algorithm, which can achieve more compact and meaningful tensor bases than GND-PCA [11, 12]. In this paper, we first apply it to statistical texture modeling of medical volumes. With LTC, medical volumes can be represented by a linear combination of bases, which are mutually independent. Furthermore, it is possible to choose the distinctive basis for classification. The proposed method was evaluated using a medical volume database. In the experiment, we compared both reconstructed results and classification results of LTC and GND-PCA. As for reconstruction results, the performance of LTC is superior to that of GND-PCA. Additionally, in the classification part, we first choose the distinctive basis based on the correlation between category labels and the coefficients of LTC basis, and then we use the selected basis for classification. The classification accuracy can be significantly improved by the use of selected distinctive basis.

This paper is organized as follows. In Section 2, a brief review of basic theory of tensor and GND-PCA is made. LTC algorithm is introduced in Section 3, and analysis is given.  Section 4 is the experimental part, and it illustrates the performance of LTC to be better than that of GND-PCA.  Section 5 summarizes the key points of this paper.

## 2. Preliminaries

 In this section, we provide a brief overview of tensor and multilinear algebra. In mathematics, multilinear algebra extends the methods of linear algebra. Just as linear algebra is built on the concept of a vector and develops the theory of vector spaces, multilinear algebra builds on the concepts of a tensor. A tensor is a multidimensional array. More formally, an *N*th-order tensor is an element of the tensor product of *N* vector spaces, each of which has its own coordinate system.

### 2.1. Tensor Definitions

As mentioned earlier, scalers are denoted by italic-shape letters, that is, (*a*, *b*,…) or (*A*, *B*,…). Bold lower case letters, that is, (**a**, **b**,…), are used to represent vectors. Matrices are denoted by bold upper case letters, that is, (**A**, **B**,…), and higher-order tensors (more than third-order tensor) are denoted by calligraphic upper case letters, that is, (*𝒜*, *ℬ*,…).

 The order of a tensor is the number of dimensions, as known as ways or modes. An *N*th-order tensor *𝒜* is defined as a multiarray with *N* indices, where *𝒜* ∈ ℝ^*I*_1_×*I*_2_×⋯×*I*_*N*_^ and ℝ is the real manifold. Elements of the tensor *𝒜* are denoted as *a*_*i*_1_⋯*i*_*n*_⋯*i*_*N*__, where 1 ⩽ *i*_*n*_⩽*I*_*n*_. The space of the *N*th-order tensor is comprised by the *N* mode subspaces. From the perspective of *𝒜*, scalars, vectors, and matrices can be seen as zeroth-order, first-order, and second-order tensors, respectively. 

The *i*th entry of a vector **a** is denoted by *a*_*i*_, element (*i*, *j*) of a matrix **A** is denoted by *a*_*ij*_, and element (*i*, *j*, *k*) of a 3rd-order tensor *𝒳* is denoted by *x*_*ijk*_. Indices typically range from 1 to their capital version; for example, *i* = 1,…, *I*. The *n*th element in a sequence is denoted by a superscript in parentheses; for example, **A** ^*n*^ denotes the *n*th matrix in a sequence.

Subarrays are formed when a subset of the indices is fixed. For matrices, these are the rows and columns. A colon is used to indicate all elements of a mode. Thus, the *j*th column of **A** is denoted by **a**_:*j*_, and the *i*th row of **A** is denoted by **a**_*i*:_.

**Figure 1:**
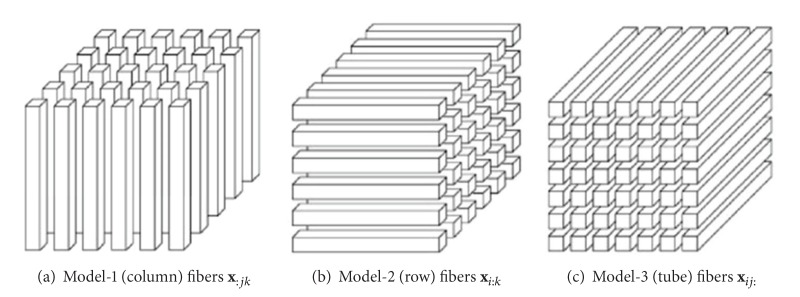
Fibers of a 3rd-order tensor.

Fibers are the higher-order analogue of matrix rows and columns. A fiber is defined by fixing every index but one. A matrix column is a mode-1 fiber and a matrix row is a mode-2 fiber. Third-order tensors have column, row, and tube fibers, denoted as **x**_:*jk*_, **x**_:*jk*_, and **x**_:*jk*_, respectively. Fibers of a Third-order tensors are shown in Figure 1.

**Figure 2:**
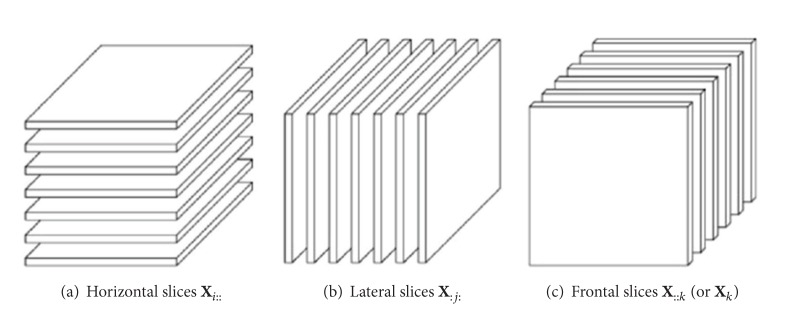
Slices of a 3rd-order tensor.

Slices are two-dimensional sections of a tensor, defined by fixing all but two indices.  Figure 2 shows the horizontal, lateral, and frontal slices of a third-order tensor *𝒳*, denoted by **X**_*i*::_, **X**_:*j*:_, and **X**_::*k*_, respectively.

The norm of a tensor *𝒳* ∈ ℝ^*I*_1_×*I*_2_×⋯×*I*_*N*_^ is the square root of the sum of the squares of all its element; that is,

(1) $$\begin{array}{c} ||𝒳||=\sqrt{\sum_{i_{1}=1}^{I_{1}}\;\sum_{i_{2}=1}^{I_{2}}\cdots \sum_{i_{N}=1}^{I_{N}}x_{i_{1}i_{2}\ldots i_{N}}^{2}}. \end{array}$$

This is analogous to the matrix Frobenius norm, which is denoted as ||**A**|| for a matrix **A**.

The inner product of two same-sized tensors *𝒳*, *𝒴* ∈ ℝ^*I*_1_×*I*_2_×⋯×*I*_*N*_^ is the sum of the products of their entries; that is,

(2) $$\begin{array}{c} \langle 𝒳,𝒴\rangle =\sum_{i_{1}=1\;}^{I_{1}}\sum_{i_{2}=1}^{I_{2}}\cdots \sum_{i_{N}=1}^{I_{N}}x_{i_{1}i_{2}\cdots i_{N}}y_{i_{1}i_{2}\cdots i_{N}}. \end{array}$$

It follows immediately that 〈*𝒳*, *𝒳*〉 = ||*𝒳*||^2^.

A *N*-order tensor *𝒳* ∈ ℝ^*I*_1_×*I*_2_×⋯×*I*_*N*_^ is rank one if it can be written as the outer product of *N* vectors; that is,

(3) $$\begin{array}{c} 𝒳=a^{\left(1\right)}\circ a^{\left(2\right)}\circ \cdot \cdot \cdot \circ a^{\left(N\right)}. \end{array}$$

**Figure 3:**
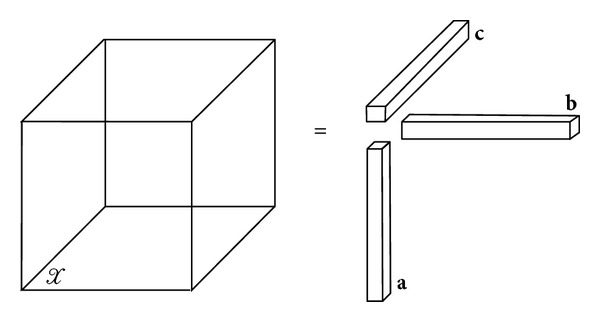
Rank-one 3rd-order tensor, *𝒳* = **a**∘**b**∘**c**.

The symbol “∘” represents the vector outer product. This means that each element of the tensor is the product of the corresponding vector elements: *x*_*i*_1_*i*_2_···*i*_*N*__ = *a* ^(1)^ *a* ^(2)^ · ··*a* ^(*N*)^, for all 1 ≤ *i*_*n*_ ≤ I_*N*_.  Figure 3 illustrates *𝒳* = **a**∘**b**∘**c**, a third-order rank-one tensor.

### 2.2. GND-PCA

 GND-PCA was proposed by Xu and Chen for statistical appearance modeling of medical volumes with few samples [10]. The medical volume is treated as a 3rd-order tensor, and the optimal subspace on each mode is calculated simultaneously by minimizing the square error between the original volumes and reconstructed ones. In the following part of this section, we will briefly review the algorithm of GND-PCA.

Given a series of the *N*-order tensors with zero-means *𝒜*_*i*_ ∈ ℝ^*I*_1_×*I*_2_×⋯×*I*_*N*_^, *i* = 1,2,…, *M*, *M* is the number of samples. We aim to get another series of low rank {*J*_1_, *J*_2_ … *J*_*N*_} tensors $\overset{\wedge }{{𝒜}_{i}}$ which accurately approximate the original tensors, where *J*_*n*_ ≤ *I*_*n*_. The new series is decomposed by the matrices **U** ^(*n*)^ ∈ ℝ^*I*_*n*_×*J*_*n*_^ with orthogonal columns according to the Turker Model [13] which is shown by

(4) $$\begin{array}{c} \overset{\wedge }{{𝒜}_{i}}\;={ℬ}_{i}\times_{1}U^{\left(1\right)}\times_{2}U^{\left(2\right)}\times \cdots \times_{n}U^{\left(n\right)}\times \cdots \times_{N}U^{\left(N\right)}, \end{array}$$

where *ℬ*_*i*_ ∈ ℝ^*J*_1_×*J*_2_×⋯×*J*_*N*_^ are the core tensors. The product operator in (4) is matrix product. Tensor *ℬ*_*i*_ will unfold to matrix in each mode and then products with orthogonal matrix in each mode. The orthogonal matrices **U** ^(*n*)^ can be determined by minimizing the cost function as

(5) $$\begin{array}{c} S=\sum_{i=1}^{M}{||{𝒜}_{i}-{ℬ}_{i}\times_{1}U^{\left(1\right)}\times_{2}U^{\left(2\right)}\times \cdots \times_{N}U^{\left(N\right)}||}^{2}. \end{array}$$

The tensor *ℬ*_*i*_ is chosen as

(6) $$\begin{array}{c} {ℬ}_{i}={𝒜}_{i}\times_{1}{U^{\left(1\right)}}^{T}\times_{2}{U^{\left(2\right)}}^{T}\times \cdots \times_{N}{U^{\left(N\right)}}^{T}. \end{array}$$

Minimization of (5) is equal to the maximization of the following equation:

(7) $$\begin{array}{c} S^{\prime }=\sum_{i=1}^{M}{||{𝒜}_{i}\times_{1}{U^{\left(1\right)}}^{T}\times_{2}{U^{\left(2\right)}}^{T}\times \cdots \times_{N}{U^{\left(N\right)}}^{T}||}^{2}. \end{array}$$

There is no close-form solution to simultaneously resolve the matrices for (4); however, the explicit solution for one matrix can be obtained if the other matrices are fixed. This is expressed by Lemma 1 and is explained later.

**Lemma 1**

Given the fixed matrices, **U** ^(1)^, **U** ^(2)^,…, **U** ^(*n*−1)^, **U** ^(*n*+1)^,…, **U** ^(*N*)^, if the columns of the matrix **U** ^(*n*)^ are selected as the first *J*_*N*_ leading eigenvectors of matrix ∑_*i*=1_^*M*^(*C*_*i*(*n*)_ · *C*_*i*(*n*)_^*T*^), where *C*_*i*(*n*)_ is the mode-n matrix of the tensor *𝒞*_*i*_ = *𝒜*_*i*_×_1_ **U** ^(1)^×_2_ **U** ^(2)^ × ⋯×_*n*−1_ **U** ^(*n*−1)^×_*n*+1_ **U** ^(*n*+1)^ × ⋯×_*N*_ **U** ^(*N*)^, the cost function *S*′ can be maximized.

The proof of Lemma 1 is given in [10], so here it will not be given again. According to Lemma 1 we can use an iteration algorithm to get the *N* optimal matrices, **U**_opt_^(1)^, **U**_opt_^(2)^,…, **U**_opt_^(*N*)^, which are able to maximize the cost function *S*′. This algorithm is summarized by Algorithm 1.

**Figure 4:**
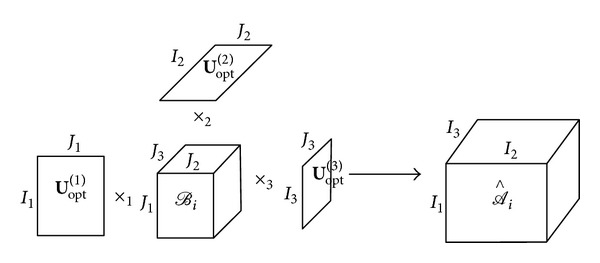
Illustration of reconstructing a third-order tensor by the principal component *ℬ*_*i*_ and the three orthogonal bases of mode subspaces **U**_opt_^(1)^, **U**_opt_^(2)^, **U**_opt_^(3)^.

Using the calculated matrices **U**_opt_^(*n*)^, *n* = 1,2,…, *N*, each of the volume *𝒜*_*i*_ are represented with least errors $\overset{\wedge }{{𝒜}_{i}}$, where $\overset{\wedge }{{𝒜}_{i}}={ℬ}_{i}\times_{1}U^{\left(1\right)}\times_{2}U^{\left(2\right)}\times \cdots \times_{n}U^{\left(n\right)}\times \cdots \times_{N}U^{\left(N\right)}$. The approximation can be illustrated by Figure 4 for the 3D case. The core tensors *ℬ*_*i*_ are the principle components.

## 3. Linear Tensor Coding

**Figure 5:**
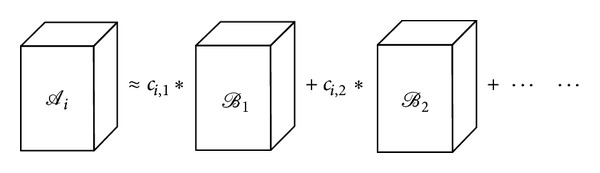
Example of representing the third-order tensor using a series of bases.

 Although GND-PCA can achieve good performance on construction of statistical appearance models for medical volumes with few samples, it still has some disadvantages. Each basis of GND-PCA is not independent, so the core tensor of the final result is still redundant. And it is difficult to understand the meaning of each basis. Thus for given a series of the *N*-order tensors with zero-means *𝒜*_*i*_ ∈ ℝ^*I*_1_×*I*_2_×⋯×*I*_*N*_^, *i* = 1,2,…, *M*, we want to find another series of bases which have mutual independence and greater discrimination to represent the original tensors. Each tensor *𝒜*_*i*_ is represented by basis: *𝒜*_*i*_ = ∑_*j*=1_ *c*_*i*,*j*_ · *ℬ*_*j*_. Here the tensor *ℬ*_*j*_ is basis which has the same size as the input tensor, and the scalar *c*_*i*,*j*_ is the coefficient of the tensor *𝒜*_*i*_.  Figure 5 illustrates the representation of one original tensor using a series of bases. 

In mathematics, the problem of getting the compact representation can be formulated as the optimization equation

(8) $$\begin{array}{c} \left({ℬ}_{1},{ℬ}_{2},\ldots \right)=\arg\;\min\sum_{i=1}^{M}||{𝒜}_{i}-\sum_{j=1}c_{i,j}\cdot {ℬ}_{j}||. \end{array}$$

Since the objective function is multiquadratic, there is no closed-form solution for this optimization. In addition, the number of bases is unfixed; hence, the optimization procedure is sensitive to initial estimation and easy to converge to local minima.

To address such problems, we have developed an algorithm: linear tensor coding algorithm (LTC) in our previous work [11, 12]. There are two important components in our algorithms; one is a local convergence to find optimized basis *ℬ*_*j*_ and the other is a global convergence to find the number of bases.

In the local parts, the GND-PCA method is applied for calculation of each basis. Inspired by (3), if we get N vectors, we can generate an *N*-order tensor. Thus when we calculate the eigenspace on each mode, we only need the first vector **u**_1_^(*i*)^ of each **U** ^(*i*)^ which is the eigenvector with the largest eigenvalue in the corresponding mode. We choose **u**_1_^(*i*)^, 1 ⩽ *i* ⩽ *N* as a set of initial estimations and the first tensor-formed base is calculated by

(9) $$\begin{array}{c} {ℬ}_{1}=u_{1}^{\left(1\right)}\circ u_{1}^{\left(2\right)}\circ \cdots \circ u_{1}^{\left(N\right)}. \end{array}$$

For each training tensor, the parameters corresponding to the first base are calculated by

(10) $$\begin{array}{c} c_{i,1}={𝒜}_{i}\times_{1}{u_{1}^{\left(1\right)}}^{T}\times_{2}{u_{1}^{\left(2\right)}}^{T}\times \cdots \times_{N}{u_{1}^{\left(N\right)}}^{T}. \end{array}$$

After getting the first base, we calculate the residual parts of each training tensor: ${\bar{𝒜}}_{i}={𝒜}_{i}-c_{i,1}\cdot {ℬ}_{1}$. The residual parts ${\bar{𝒜}}_{i}$ are used instead of *𝒜*_*i*_. Then the previous step was repeated, to calculate the basis one by one. The process to find a series of bases is a greedy approach to approximate the original tensors.

A global convergence is worked to find a number of bases. Recalling (8), we assign a threshold *r*. The process ends after finding the *J* basis, when the sum of norms of the residual tensors is below *r*, as shown in (11). Then each tensor data is represented with a group of coefficients with the benefit of the obtained basis. Consider

(11) $$\begin{array}{c} \mathrm{norm}\left(\sum_{i=1}^{M}||{𝒜}_{i}-\sum_{j=1}^{J}c_{i,j}*{ℬ}_{j}||\right)\leq r. \end{array}$$

The global process converges to a local minima, and thus there is no guarantee that there will be a global one. As this is a greedy approach, it suffers from the shortcoming that previous decisions are not reevaluated as the process unfolds. However, this specific greedy rule has a critical feature which makes it useful for tensor coding. Note that the optimization approach converges to a local minima in general, but in the case we just choose one base in LTC, one obtains GND-PCA for representing the data with a core tensor of which the rank of each mode is 1. So LTC can be considered as an extension of GND-PCA. The algorithm is shown in Algorithm 2.

## 4. Experimental Results

**Figure 6:**
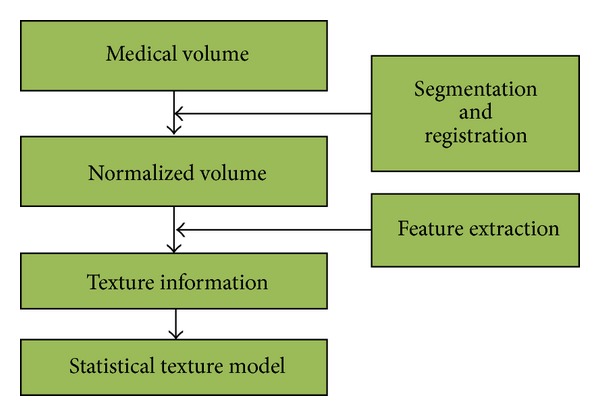
The flow chart of our experiment.

 The proposed method is evaluated by using a liver database. In this database, there are 10 normal healthy ones and 10 abnormal ones. The size of each sample is 256 × 256 × 79. The flow chart of our experiment is shown in Figure 6. 

In order to remove shape variations, we apply a nonrigid transformation based on mathematical forms for morphing all the datasets to a same shape. Any nonrigid registration technique can be described by three components: a transformation which relates the target and source images, a similarity measure which measures the similarity between target and source image, and an optimization which determines the optimal transformation parameters as a function of the similarity. Additionally, we do not need to assume the physical parameters, which are difficult to guess in practice. Hence, we adopted the mathematical nonrigid transformation in our research. For the detailed process, please refer to [14].

**Figure 7:**
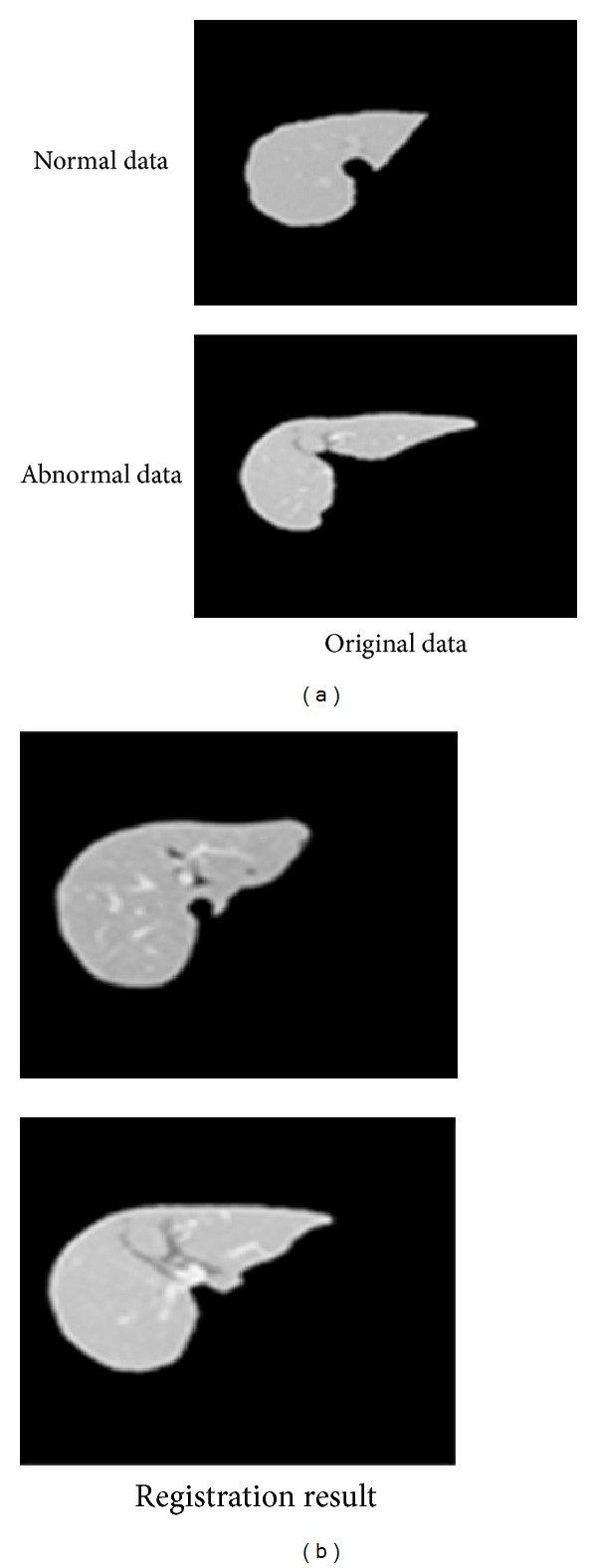
Original data and morphed data, respectively. The first column is original data and the second column is morphed data.

The pretreated database is assigned as original database, and shape-normalized samples are assigned as morphed database.  Figure 7 shows some original data and morphed data, respectively. The first column is original data and the second column is morphed data. The first row is one sample of the normal ones, and the second row is one sample of the abnormal ones. This illustrates that all the samples have familiar shapes, so the shape information does not effect experimental results.

Because we want to build a statistical texture model, each data can be represented by

(12) $$\begin{array}{c} {𝒜}_{i}=ℳ+\sum_{k=1}^{K}c_{i,k}*{ℬ}_{k}. \end{array}$$

Here *ℳ* is the mean texture and *c*_*i*,*k*_ are the coefficients. Supposing the coefficients *c*_*i*,*k*_ to follow Gaussian distribution, we can estimate the mean *m*_*k*_ and derivation *λ*_*k*_^2^.

By adjusting the parameter, we can construct a novel ensemble by

(13) $$\begin{array}{c} \tilde{𝒜}=ℳ+\tilde{c_{k}}*{ℬ}_{k}. \end{array}$$

**Figure 8:**
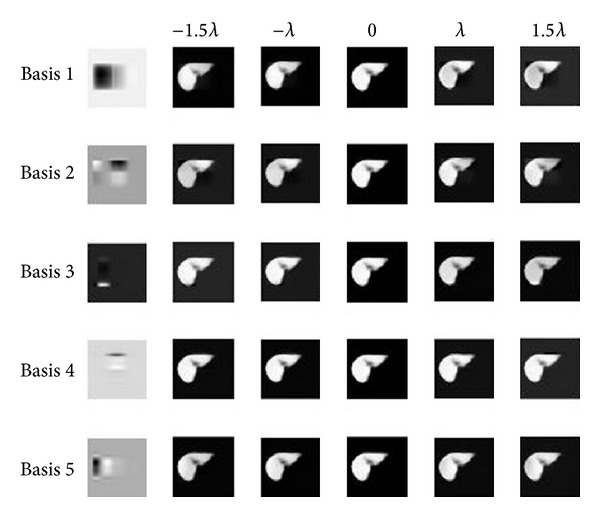
The slices of novel ensembles described by the first five bases, respectively. It changes the value of coefficients from −1.5*λ*_1_ to 1.5*λ*_1_ of each basis.

**Figure 9:**
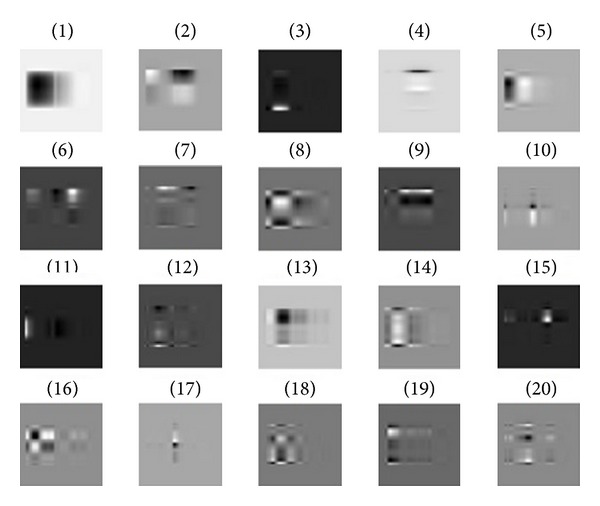
The first twenty bases. It illustrates that each basis can represent a local feature.

Here $\tilde{c_{k}}$ is adjusted coefficient, $-2\lambda_{k}⩽\tilde{c_{k}}⩽2\lambda_{k}$.  Figure 8 illustrates the slices of novel ensembles described by the first five bases, respectively. They demonstrate that while changing the value of first coefficients from −1.5*λ*_1_ to 1.5*λ*_1_, the intensity of left part has obviously changed, and the second basis mainly has effect on the right corner of the slice. Furthermore, Figure 9 shows the first twenty bases; it illustrates that different bases can have effect on different parts. Thus we can change the local intensity of slice through change the coefficient of basis.

**Figure 10:**
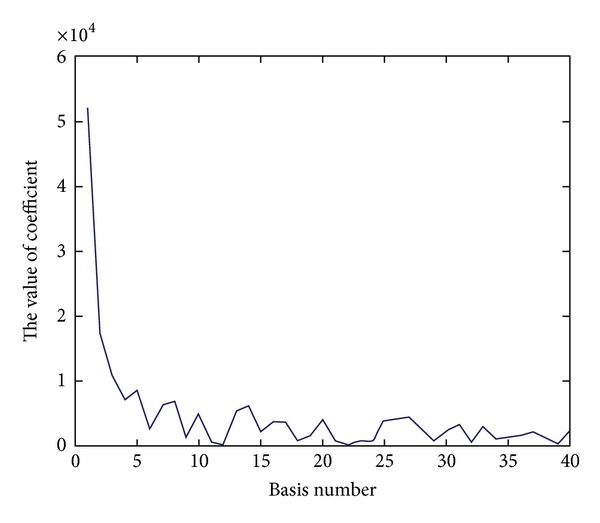
The coefficient of each basis for LTC.

Figure 10 shows the values of coefficient when the number of bases is different for LTC. It illustrates that the first several values are obviously larger than the other ones. Because of this, the volume can be reconstructed by less bases than GND-PCA.

**Figure 11:**
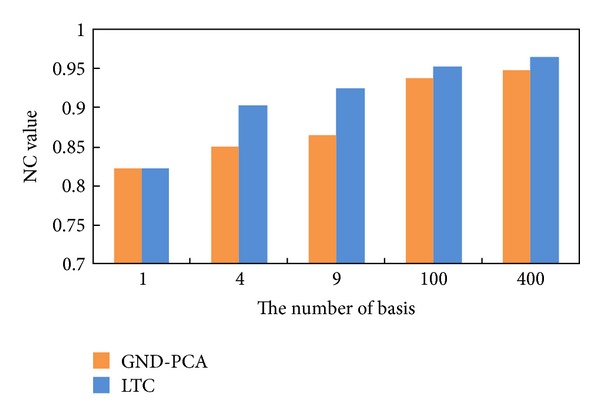
Reconstruction accuracy versus number of basis.

Figure 11 shows the graph of normalized correlation between original volume and reconstructed volume for different number of bases.The value of normalized correlation is between 0 and 1. The more similar the two volumes are, the larger its value is. The result in Figure 11 illustrates that the original volume can be better reconstructed by LTC when the number of bases is the same as GND-PCA.

**Figure 12:**
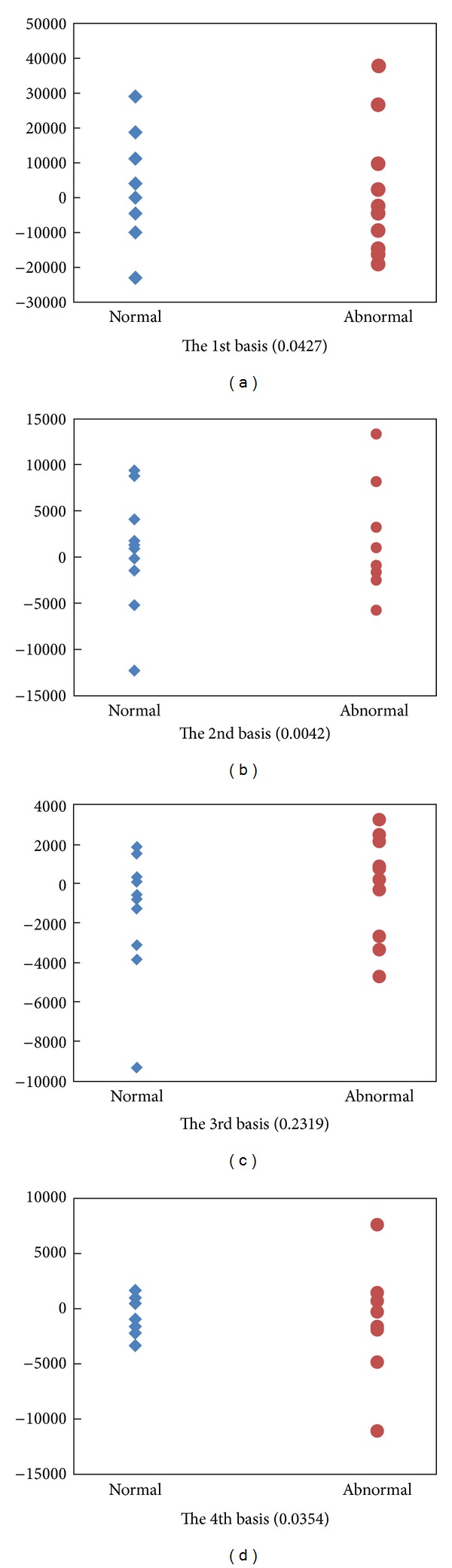
The distribution of coefficients of the first four bases. The blue one represents the normal liver and the red one represents the abnormal liver. The number in the brackets is the correlation coefficients.

 For classification, the coefficients are used as the feature; SVM and KNN are utilized as classifiers, respectively. For LTC, we trained 1200 bases, and for GND-PCA, the size of core-tensor is 20 × 20 × 3. And we used leave one out method to do the classification.  Figure 12 shows the distribution of coefficients of the first four bases of LTC. The blue ones are the normal livers, and the red ones are abnormal livers. The number in the brackets is the correlation coefficient which is calculated by

(14) correlation=∑in(ci−c−)(li−l−)∑in(ci−c−)2(li−l−)2.

**Figure 13:**
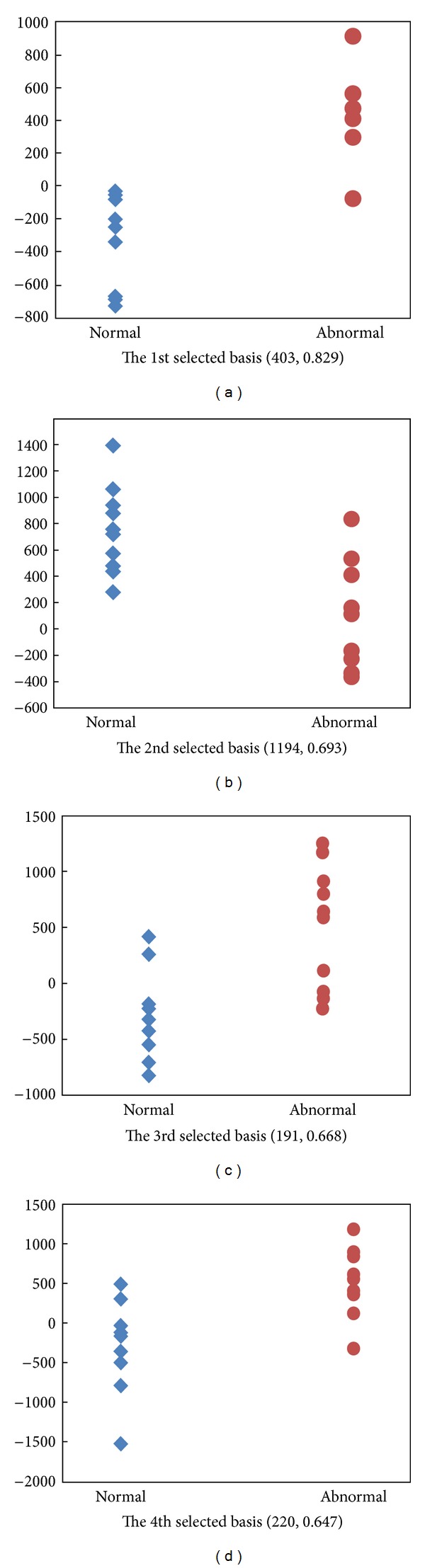
The distribution of coefficients of the first four basis chosen through correlation coefficients. The blue ones are the normal livers, and the red ones are abnormal livers. The first number in the bracket is the position of basis in the original basis set and the second number in the bracket is the correlation coefficient.

Here, *n* is the number of samples, *c*_*i*_ is the coefficient of one fixed basis of LTC, and *l*_*i*_ ∈ {−1,1}, 1 ≤ *i* ≤ *n*, is the label of the samples. In our experiments, −1 represents normal liver, and 1 represents abnormal liver. For each basis, we can get a correlation coefficient. From the correlation coefficients in Figure 12, it illustrates that it is difficult for classification if using these basis because the correlation coefficients are so small. Thus, we firstly chose the basis using the correlation coefficients. But GND-PCA cannot choose basis because the core tensor is directly used for classification.  Figure 13 shows the coefficients of the first four bases chosen through correlation coefficients. The blue ones are the normal livers, and the red ones are abnormal livers. The first number in the bracket is the position of basis in the original basis set and the second number in the bracket is the correlation coefficient of corresponding basis.

Table 1 is the classification accuracy using different classifiers. Before choosing bases, we used all the basis for classification. We can see that the classification accuracies of LTC and GND-PCA are both bad. Then we choose the first one hundred bases which have greater correlation coefficients for classification. The classification accuracy is obviously improved. 

## 5. Conclusion

 In this paper, we describe a statistical texture modeling method for medical volumes which is known as LTC. LTC is an extension of GND-PCA. The medical volume such as the volume of the liver is represented by a linear combination of bases which have the same size as the tensor. Each basis is mutual independence and more discriminate than that of GND-PCA. In our experiments, we compared both reconstructed results and classification results of LTC and GND-PCA. As for reconstruction results, the performance of LTC is superior to that of GND-PCA. Additionally, in the classification part, we firstly chose the distinctive basis through the correlation between category labels and the coefficients of basis of LTC. And then we use the selected basis for classification. The classification accuracy was significantly improved by the use of selected distinctive basis. Future work will involve testing our method with more data sets for classification and using our method in practical applications.

## Figures and Tables

**Algorithm 1:**
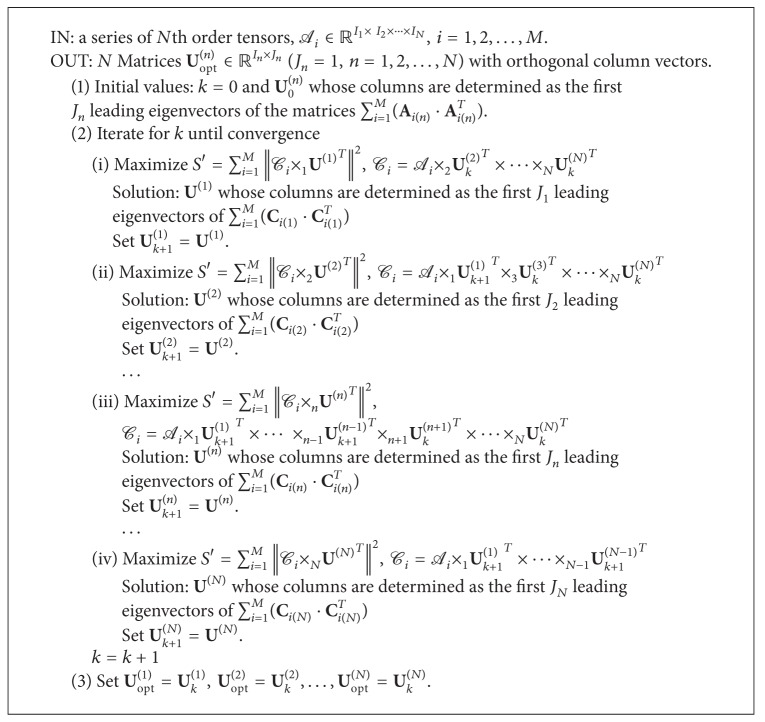
GND-PCA.

**Algorithm 2:**
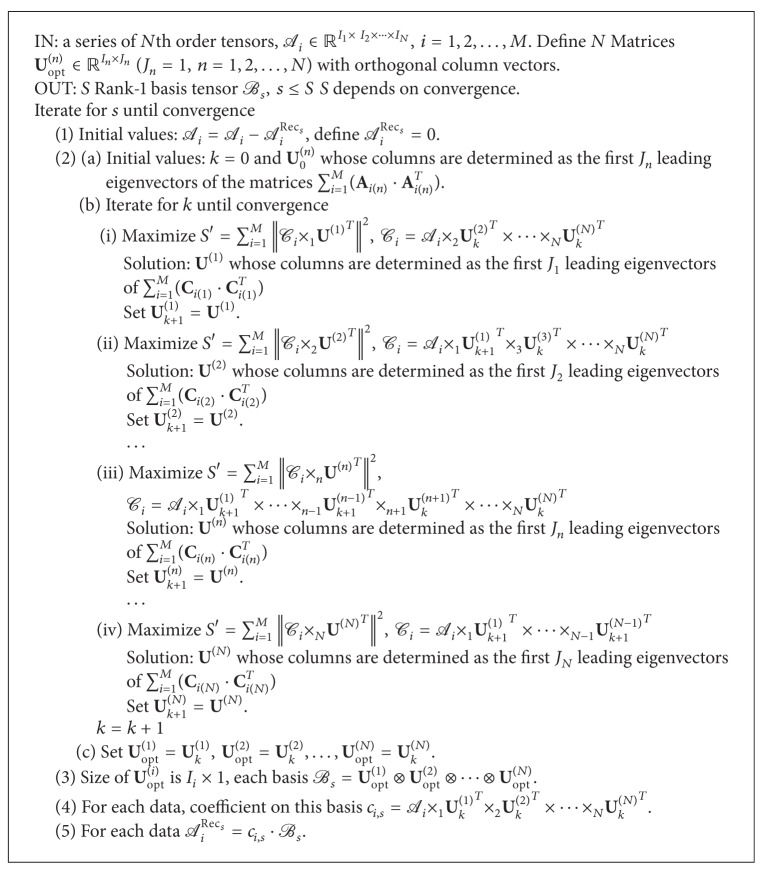
Iteration algorithm of LTC.

**Table 1:** The classification accuracy of GND-PCA and LTC.

	SVM	KNN
GND-PCA	7/20	10/20
LTC with all 1200 bases	7/20	9/20
LTC with 100 selected bases	19/20	19/20
